# A simple method for getting standard error on the ratiometric calcium estimator

**DOI:** 10.1016/j.mex.2021.101548

**Published:** 2021-10-22

**Authors:** Simon Hess, Christophe Pouzat, Peter Kloppenburg

**Affiliations:** a Institute for Zoology, Biocenter and Cologne Excellence Cluster in Aging Associated Diseases (CECAD), University of Cologne, Cologne, Germany; b IRMA, Strasbourg University and CNRS UMR 7501, Strasbourg, France

**Keywords:** Calcium measurements, Fura-2, Propagation of uncertainty, Propagation of errors, Monte-Carlo method, Reproducible research

## Abstract

The ratiometric fluorescent calcium indicator Fura-2 plays a fundamental role in the investigation of cellular calcium dynamics. Despite of its widespread use in the last 30 years, only one publication (Joucla et al., 2010)) proposed a way of obtaining confidence intervals on fitted calcium dynamic model parameters from single ‘calcium transients’. Shortcomings of this approach are its requirement for a ‘3 wavelengths’ protocol (excitation at 340 and 380 nm as usual plus at 360 nm, the isosbestic point) as well as the need for an autofluorence / background fluorescence model at each wavelength. Here, we propose a simpler method that eliminates both shortcommings:

1. a precise estimation of the standard errors of the raw data is obtained first,
2. the standard error of the ratiometric calcium estimator (a function of the raw data values) is derived using both the propagation of uncertainty and a Monte-Carlo method.

Once meaningful standard errors for calcium estimates are available, standard errors on fitted model parameters follow directly from the use of nonlinear least-squares optimization algorithms.

## Graphical abstract

How to get error bars on the ratiometric calcium estimator? The two measurements areas (region of interest, ROI, on the cell body and background measurement region, BMR, outside of the cell) are displayed on the frame corresponding to one actual experiment. Two measurements, one following an excitation at 340 nm and the other following an excitation at 380 nm are performed (at each ’time point’) from each region. The result is a set of four measures: adu${}_{340}$ (from the ROI), adu${}_{340,B}$ (from the BMR), adu${}_{380}$ and adu${}_{380,B}$.. The fact that the measurements as well as the subsequent quantities derived from them are random variable realization is conveyed throughout the figure by the use of Gaussian probability densities. The densities from the BMR are ’tighter’ because there are much more pixels in the BMR than in the ROI (the standard deviations of the densities shown on this figure have been enlarged for clarity, but their relative size has been preserved, the horizontal axis in black always starts at 0). The key result of the paper is that the standard deviation of the four Gaussian densities corresponding to the raw data (bottom of the figure) can be reliably estimated from the data alone, *eg*$\sigma_{380}\approx \sqrt{G\;adu_{380}+V}$, where V is the product of the CCD chip gain squared by the number of pixels in the ROI by the CCD chip read-out variance. The paper explains how to compute the standard deviation of the derived distributions obtained at each step of the calcium concentration estimation.

**Figure:**
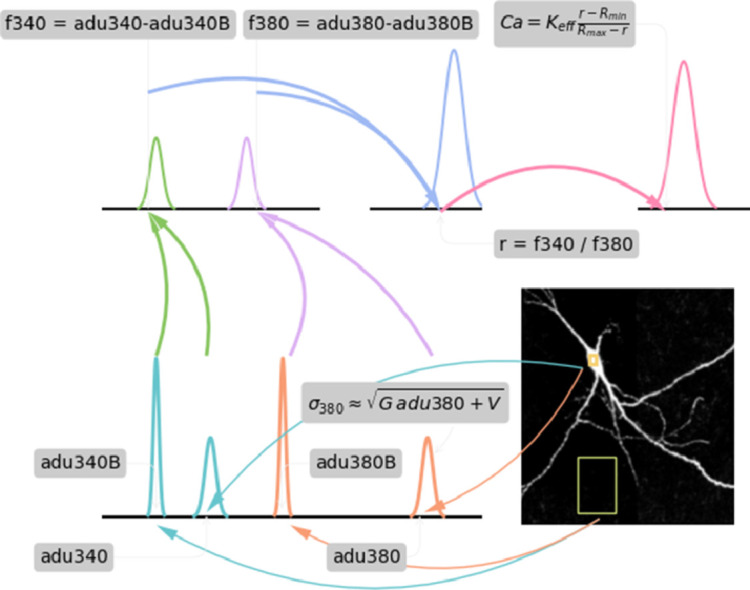

Specifications table

**Table:** 

Subject Area:	Neuroscience
More specific subject area:	*Intracellular calcium dynamics*
Method name:	Simple and quick *standard error on the ratiometric calcium estimator*
Name and reference of original method:	Sébastien Joucla, Andreas Pippow, Peter Kloppenburg and ChristophePouzat (2010) Quantitative estimation of calcium dynamics from ratiometric measurements: A direct,non-ratioing, method Journal of Neurophysiology 103: 1130-1144
Resource availability:	Python codes, data and all the information required to reproduce themanuscript results can be found on GitLab: https://gitlab.com/c_pouzat/gettingse-on-ratiometric-ca-estimator

## Method overview

### Rational

Since its introduction by Grynkiewicz et al. [2], the ratiometric indicator Fura-2 has led to a revolution in our understanding of the role of calcium ions (Ca${}^{2+}$) in neuronal and cellular function. This indicator provides a straightforward estimation of the free Ca${}^{2+}$ concentration ([Ca${}^{2+}$]) in neurons and cells with a fine spatial and time resolution. The experimentalist must determine a ‘region of interest’ (ROI) within which the [Ca${}^{2+}$] can be assumed to be uniform and is scientifically relevant. Fluorescence must be measured following excitation at two different wavelengths: typically around 340 and 380 nm; and, since cells exhibit autofluorescence or ‘background fluorescence’ at those wavelengths, the measured fluorescence intensity is made of two sources: the Fura-2 linked fluorescence and the autofluorescence. The measured intensity within the ROI is therefore usually corrected by subtrating from it an estimation of the autofluorescence intensity obtained from simultaneous measurements from a ‘background measurement region’ (BMR); that is, a nearby region where there is no Fura-2. At a given time the experimentalist will therefore collect a fluorescence intensity measurement from the ROI at 340 and 380 nm; we are going to write adu${}_{340}$ and adu${}_{380}$ these measurements, where ‘adu’ stands for ‘analog to digital unit’ and corresponds to the raw output of the fluorescence measurement device, usually a charge-coupled device (CCD); if the experimentalist is careful not to saturate the sensor, the adu count is proportional to the number of photo-electrons present in the pixel, or in the group of pixels when on-chip binning is used, at the end of the exposure period. The experimentalist will also collect intensity measurements from the BMR, measurements that we are going to write adu${}_{340,B}$ and adu${}_{380,B}$. If P CCD pixels make the ROI and P${}_{B}$ pixels make the BMR and if the illumination time at 340 nm is ${\text{T}}_{340}$, while the illumination time at 380 nm is ${\text{T}}_{380}$ (both times are measured in s), the experimentalist starts by estimating the fluorescence intensity per pixel per time unit following an excitation by a light flash of wavelengths λ (λ=340 or 380 nm) as:

(1) $$f_{\lambda }=\frac{1}{{\text{T}}_{\lambda }}\left(\frac{{\text{adu}}_{\lambda }}{\text{P}}-\frac{{\text{adu}}_{\lambda ,\text{B}}}{{\text{P}}_{\text{B}}}\right)\;,\;\text{with}\;\lambda =340\;\text{or}\;380\;\text{nm}\;,$$

where an assumption of autofluorescence uniformity is implicitly made. The following ratio is then computed:

(2) $$r=\frac{f_{340}}{f_{380}}\;.$$

This is an important and attractive feature of the method as well as the origin of its name. Since only ratios are subsequently used, geometric factors like the volume of the Fura loaded region under the ROI do not need to be estimated.

The *estimated* [Ca${}^{2+}$] that we will write $\hat{\text{Ca}}$ for short (the ’$\hat{\;}$’ sign is used for marking estimated values) is then obtained, following [2, Eq. (5), p. 3447], with:

(3) $$\hat{\text{Ca}}=K_{eff}\;\frac{r-R_{\text{min}}}{R_{\text{max}}-r}\;,$$

where $K_{eff}$ (measured in μM), $R_{\text{min}}$ and $R_{\text{max}}$ are calibrated parameters (the last two parameters are ratios and are dimensionless). $R_{\text{min}}$ is the ratio (Eq. (2)) observed in the absence of calcium, while $R_{\text{max}}$ is the ratio observed with a saturating concentration. $K_{eff}$ is the calcium concentration at which the ratio is half way between $R_{\text{min}}$ and $R_{\text{max}}$. If a set of experiments is performed on a given cell type with the same batch of Fura, as in the companion paper [3], the calibration errors on these three parameters will be the same for each experiment. If different cell types are considered and/or different Fura batches are used, the calibration errors should be taken into account before making comparison of estimated calcium dynamics parameters (see [1] for discussion).

If we now want to rigorously fit [Ca${}^{2+}$] dynamics models to sequences of $\hat{\text{Ca}}$, we need to get *standard errors*, $\sigma_{\hat{\text{Ca}}}$, on our estimates. This is where the ratiometric method gets ‘more involved’, at least if we want standard errors from a single transient as opposed to a mean of many transients. We typically work (*e.g.* [1], [3]) in a setting, using the so called ‘added buffer approach’, where we cannot get more than a single transient in given conditions since Fura is constantly diffusing into the recorded cell modifying thereby the time constant of calcium transients. It is worth pointing out that there is a more general interest in obtaining standard errors from a single transient: getting these fluorescence measurements requires shining UV light on the neurons we are recording from and generates photodamage. Despite the ubiquity of ratiometric measurements in neuroscience and cell physiology, we are aware of a single paper–by some of us [1]–where the ’standard error question’ was directly addressed. The method proposed in [1] requires a 3 wavelengths protocol: measurements at 340, 380 *and* 360 (the isosbestic wavelength) nm; it drops, so to speak, the above advantage of working with a ratiometric estimator since it fits directly the adu${}_{340}$ and adu${}_{380}$ data (at the cost of estimating some geometry related parameters) and it requires a model of the autofluorescence dynamics if the latter is not stationnary. It therefore requires a slightly more complicated ’3 wavelengths’ recording protocol as well as a more involved fitting procedure. The dataset of the companion paper [3] exhibits a clear but reversible autofluorescence rundown that cannot be ignored since autofluorescence accounts for half of the signal in the ROI. Rather that constructing / tailoring the accurate enough autofluorence models required by the ’direct approach’ of [1] we looked for an alternative method providing standard errors for the ratiometric estimator.

## Ratiometric estimator variance

### Fluorescence intensity

As detailed in [1], [2], the fluorescence intensities giving rise to the adu${}_{340}$, adu${}_{340,B}$, adu${}_{380}$ and adu${}_{380,B}$ signals can be written as:

(4) $$I_{340}=\left\{\frac{{\left[Fura\right]}_{total}\;\phi }{K_{Fura}+\left[Ca^{2+}\right]}\left(R_{\text{min}}\;K_{eff}+R_{\text{max}}\left[Ca^{2+}\right]\right)+F_{340B}\right\}\;T_{340}\;P\;,$$

(5) $$I_{340B}=F_{340B}\;T_{340}\;P_{B}\;,$$

(6) $$I_{380}=\left\{\frac{{\left[Fura\right]}_{total}\;\phi }{K_{Fura}+\left[Ca^{2+}\right]}\left(K_{eff}+\left[Ca^{2+}\right]\right)+F_{380B}\right\}\;T_{380}\;P\;,$$

(7) $$I_{380B}=F_{380B}\;T_{380}\;P_{B}\;,$$

where $F_{\lambda B}$ is the autofluorescence intensity per pixel per time unit at wavelength λ, $K_{Fura}$ is the Fura dissociation constant (a calibrated parameter measured in μM), ${\left[Fura\right]}_{total}$, is the total (bound plus free) concentration of Fura in the cell (measured in μM) and ϕ is an experiment specific parameter (measured in 1/μM/s) lumping together the quantum efficiency, the neurite volume, etc (see [1] for details).

### Recorded signals adu${}_{340}$, adu${}_{340,B}$, adu${}_{380}$ and adu${}_{380,B}$

As detailed and discussed in [1], [4], the signal adu${}_{\lambda }$ recorded with a CCD chip whose gain is G and whose read-out variance is $\sigma_{read-out}^{2}$ can be modeled as the realization of a Gaussian random variable ADU${}_{\lambda }$ with parameters:

(8) $$\mu_{ADU_{\lambda }}=G\;I_{\lambda }\;,$$

(9) $$\sigma_{ADU_{\lambda }}^{2}=G\;\mu_{ADU_{\lambda }}+G^{2}\;P\;\sigma_{read-out}^{2}\;,$$

with the obvious adaptation when dealing with the BMR signal: $I_{\lambda }$ is replaced by $I_{\lambda B}$ and P is replaced by $P_{B}$. Parameters G and $\sigma_{read-out}^{2}$ are CCD chip parameters provided by the manufacturer. Calibration procedures are discussed in [1], [4] and a comprehensive example with data and codes can be found in [5]. Our experience is that the values provided by manufacturers are good starting points; the user calibrated read-out noise is sometime slightly larger than the one specified by the manufacturer.

### Variance estimates for adu${}_{340}$, adu${}_{340,B}$, adu${}_{380}$ and adu${}_{380,B}$

So, to have the variance of $ADU_{\lambda }$ we need to know $I_{\lambda }$ and for that we need to know $\left[Ca^{2+}\right]$ (Eqs. (4) and (6)) precisely what we want to estimate. But the expected value of $ADU_{\lambda }$ is $G\;I_{\lambda }$ (Eq. (8)), we can therefore use as a first approximation the observed value $adu_{\lambda }$ of $ADU_{\lambda }$ as a guess for $G\;I_{\lambda }$, so in Eq. (9) we plug-in $adu_{\lambda }$ for $G\;I_{\lambda }$, leading to:

(10) $${\hat{\sigma }}_{ADU_{\lambda }}^{2}=G\;adu_{\lambda }+G^{2}\;P\;\sigma_{read-out}^{2}\approx \sigma_{ADU_{\lambda }}^{2}\;.$$

In other words, we will use the observed $adu_{\lambda }$ as if it were the actual fluorescence intensity times the CCD chip gain, $ADU_{\lambda }=G\;I_{\lambda }$, in order to estimate the variance. In doing so we will sometime slightly underestimate the actual variance (when the observed $adu_{\lambda }$ turns out to be smaller than $ADU_{\lambda }$) and sometime slightly overestimate it (when the observed $adu_{\lambda }$ turns out to be larger than $ADU_{\lambda }$). Since we are going to combine many such approximations, we expect–and we will substantiate this claim in Section 3–that overall the under-estimations will be compensated for by the over-estimations.

### Variance estimate for $\hat{Ca}$

Now that we have a ${\hat{\sigma }}_{ADU_{\lambda }}^{2}$ we can work with – that is, an estimate from the data alone –, we want to get ${\hat{\sigma }}_{r}^{2}$ (Eq. (2)) and ${\hat{\sigma }}_{\hat{Ca}}^{2}$. We can either use the propagation of uncertainty (also referred to as *error propagation, compounding of errors* or *delta method*) [6], [7] together with Eqs. (2) and (3), or a ’quick’ Monte Carlo approach. We drop any explicit time index in the sequel in order to keep the equations more readable, but it should be clear that such variance estimates have to be obtained for each sampled point.

#### Propagation of uncertainty

This method requires, in the general case, an assumption of ‘small enough’ standard error since it is based on a first order Taylor expansion (see Section Appendix A for details). It leads first to the following expression for the variance, ${\hat{\sigma }}_{f_{\lambda }}^{2}$, of $f_{\lambda }$ in Eq. (1):

(11) $${\hat{\sigma }}_{f_{\lambda }}^{2}\approx \frac{1}{T_{\lambda }^{2}}\;\left(\frac{{\hat{\sigma }}_{ADU_{\lambda }}^{2}}{P^{2}}+\frac{{\hat{\sigma }}_{ADU_{\lambda }B}^{2}}{P_{B}^{2}}\right)\;.$$

The variance ${\hat{\sigma }}_{r}^{2}$ of r in Eq. (2) is then:

(12) $${\hat{\sigma }}_{r}^{2}\approx \frac{1}{f_{380}^{2}}\;\left({\hat{\sigma }}_{f_{340}}^{2}+r^{2}\;{\hat{\sigma }}_{f_{380}}^{2}\right)$$

and the variance ${\hat{\sigma }}_{\hat{Ca}}^{2}$ of $\hat{Ca}$ in Eq. (3) is:

(13) $${\hat{\sigma }}_{\hat{Ca}}^{2}\approx {\left(\frac{K_{eff}}{R_{\text{max}}-r}\right)}^{2}\;{\left(1+\hat{Ca}\right)}^{2}\;{\hat{\sigma }}_{r}^{2}\;.$$

#### A remark on ${\hat{\sigma }}_{\hat{Ca}}^{2}$ behavior

**Fig. 1:**
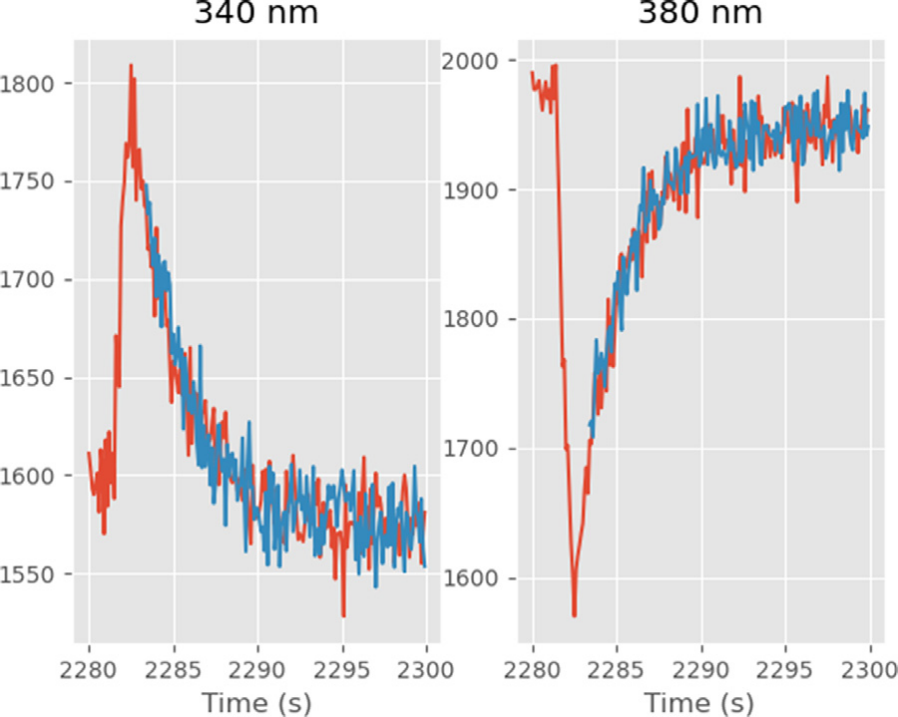
Observed (red) and simulated (blue) ADU at 340 (left) and 380 nm (right) for the first transient (only the late phase of the transient was simulated).

**Fig. 2:**
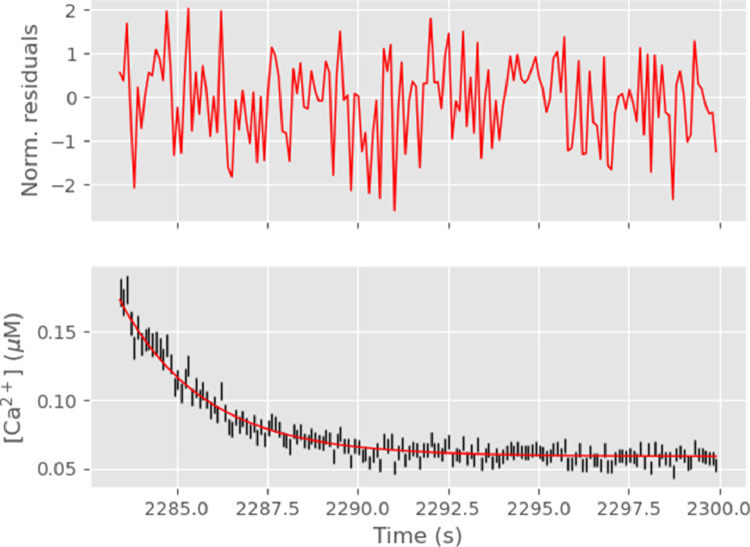
Top: Simulated ratiometric estimator - ‘actual’ [Ca${}^{2+}$] divided by ratiometric estimator standard error (if everything goes well we should see draws from a standard normal distribution); bottom: Simulated ratiometric estimator (with error bars given by the standard error) in black and ‘actual’ [Ca${}^{2+}$] in red.

The last three Eqs. (11)–(13) can be used together with Eqs. (8) and (9) to understand why ${\hat{\sigma }}_{\hat{Ca}}^{2}$ will increase with the calcium concentration and therefore why a *weighted* nonlinear least-square procedure is required [8], [9], [10] in order to get proper confidence intervals on calcium dynamics model parameters. Eq. (9) tells us that the variance of the raw signals is an increasing linear function of their means. When the calcium concentration increases, the recorded signal at 340 nm increases while the one at 380 nm decreases (Fig. 1). So according to Eq. (11), ${\hat{\sigma }}_{f_{340}}^{2}$ increases while ${\hat{\sigma }}_{f_{380}}^{2}$ decreases in proportion to [Ca${}^{2+}$]. From Eq. (12) we see that ${\hat{\sigma }}_{r}^{2}$ also increases since ${\hat{\sigma }}_{f_{340}}^{2}$ does increase and $r^{2}\;{\hat{\sigma }}_{f_{380}}^{2}$ is roughly proportional to $f_{340}^{2}/f_{380}$ and increases. Then from Eq. (13) we see that r is getting closer to $R_{\text{max}}$, therefore the denominator is decreasing, while we just argued that ${\hat{\sigma }}_{r}^{2}$ increases. Together, the two imply that ${\hat{\sigma }}_{\hat{Ca}}^{2}$ is an increasing function of [Ca${}^{2+}$]. This can be seen on the bottom panel of Fig. 2 where the error bars on the left side (corresponding to larger [Ca${}^{2+}$]) are about twice as large as the ones on the right side (corresponding to smaller [Ca${}^{2+}$]).

#### Monte-Carlo method

Here we draw, k quadruple of vectors

$$\left(adu_{340}^{\left[j\right]},adu_{340B}^{\left[j\right]},adu_{380}^{\left[j\right]},adu_{380B}^{\left[j\right]}\right)\;,\;j=1,\ldots ,k\;,$$

from four independent Gaussian distributions of the general form:

(14) $$adu_{\lambda }^{\left[j\right]}=adu_{\lambda }+z_{\lambda }^{\left[j\right]}\;{\hat{\sigma }}_{ADU_{\lambda }}\;,$$

where $adu_{\lambda }$ is the observed value and $z_{\lambda }^{\left[j\right]}$ is drawn from a standard normal distribution. We then plug-in these quadruples into Eq. (1) leading to k couples:

$$\begin{array}{cc} f_{340}^{\left[j\right]} & =\frac{1}{{\text{T}}_{340}}\left(\frac{adu_{340}^{\left[j\right]}}{\text{P}}-\frac{adu_{340B}^{\left[j\right]}}{{\text{P}}_{\text{B}}}\right)\;,\\ f_{380}^{\left[j\right]} & =\frac{1}{{\text{T}}_{380}}\left(\frac{adu_{380}^{\left[j\right]}}{\text{P}}-\frac{adu_{380B}^{\left[j\right]}}{{\text{P}}_{\text{B}}}\right)\;,\;j=1,\ldots ,k\;. \end{array}$$

These k couples are ‘plugged-in Eq. (2)’ leading to k $r^{\left[j\right]}$:

$$r^{\left[j\right]}=\frac{f_{340}^{\left[j\right]}}{f_{380}^{\left[j\right]}}\;\;j=1,\ldots ,k\;,$$

before plugging in the latter into Eq. (3) to get k ${\hat{Ca}}^{\left[j\right]}$:

$${\hat{Ca}}^{\left[j\right]}=K_{eff}\frac{r^{\left[j\right]}-R_{\text{min}}}{R_{\text{max}}-r^{\left[j\right]}}\;\;j=1,\ldots ,k\;.$$

The empirical variance of these simulated observations will be our ${\hat{\sigma }}_{\hat{Ca}}^{2}$:

(15) $${\hat{\sigma }}_{\hat{Ca}}^{2}=\frac{1}{k-1}\sum_{j=1}^{k}{\left({\hat{Ca}}^{\left[j\right]}-{\hat{Ca}}_{•}\right)}^{2}\;,\;\;\text{where}\;\;{\hat{Ca}}_{•}=\frac{1}{k}\sum_{j=1}^{k}{\hat{Ca}}^{\left[j\right]}\;.$$

Since the Monte-Carlo method requires milder assumptions (the variances do not have to be small) and is easy to adapt, we tend to favor it; to be on the safe side, users can use both methods and, if they disagree, plot a histogram of the ${\hat{Ca}}^{\left[j\right]}$ to make sure that the discrepancy source is the non-normality of the latter.

### Comment

The present approach based on a ${\hat{\sigma }}_{\hat{Ca}}^{2}$ estimation is slightly less rigorous than the ‘direct approach’ of [1] but it is far more flexible since it does not require an independent estimation / measurement of ${\left[Fura\right]}_{total}$. In line with the discussion following Eq. (3), in the companion paper [3] we chose to consider the calibrated parameters $K_{eff}$, $R_{\text{min}}$ and $R_{\text{max}}$ as fixed.

## Empirical validation

### Rational

Eqs. (4)–(7), together with Eqs. (8) and (9) can be viewed as a data generation model. This means that if we choose model parameters values as well as an arbitrary [Ca${}^{2+}$] time course, we can simulate measurements (adu) at both wavelengths in the ROI as well as in the BMR. We can then use these simulated adu exactly as we used the actual data, namely get $r(t_{i})$ (Eq. (2)) and $\hat{Ca}\left(t_{i}\right)$ (Eq. (3)) as well as the (squared) standard errors ${\hat{\sigma }}_{\hat{Ca}}^{2}\left(t_{i}\right)$ (Section 2.4).

Now if the ${\hat{\sigma }}_{\hat{Ca}}^{2}\left(t_{i}\right)$ are good approximations for the actual but unknown $\sigma_{Ca}^{2}\left(t_{i}\right)$, the distribution of the *normalized residuals*:

$$\frac{\hat{Ca}\left(t_{i}\right)-Ca\left(t_{i}\right)}{{\hat{\sigma }}_{\hat{Ca}}\left(t_{i}\right)}\;,$$

should be very close to a standard normal distribution. *This is precisely what we are going to check*.

### Simulated data

We are going to use the first transient of dataset DA_121219_E1 of the companion paper [3]. The ‘static’ parameters – that is the parameters not link to the calcium dynamics – used for the simulation are the actual experimental parameters rounded to the third decimal (Table 1).

**Table 1:** ’Static’ parameters used for the simulation.

Parameter	Value
$R_{\text{min}}$	0.147
$R_{\text{max}}$	1.599
$K_{eff}$	1.093 (μM)
$K_{Fura}$	0.225 (μM)
${\left[Fura\right]}_{total}\phi$	1.89e+05 ($s^{-1}$)
$T_{340}$	0.01 (s)
$T_{380}$	0.003 (s)
P	3
$P_{B}$	448
G	0.146
$\sigma_{read-out}^{2}$	268.96
$F_{340B}$	189512 ($s^{-1}$)
$F_{380B}$	711589 ($s^{-1}$)

The simulated calcium dynamics is a monoexponential decay mimicking the tail of the transient:

$$Ca\left(t\right)=Ca_{0}+\left\{ \begin{array}{cc} 0 & \text{if}\;t<t_{0}\\ \delta \exp(-\left(t-t_{0}\right)/\tau ) & \text{if}\;t\geq t_{0} \end{array}\right.$$

and the parameter values (Table 2) are just a rounded version of the fitting procedure output (see companion paper [3]).

**Table 2:** Calcium dynamics parameters used for the simulation. Time 0 is when seal is obtained.

Parameter	Value
$t_{0}$	2283.415 (s)
$Ca_{0}$	0.059 (μM)
δ	0.114 (μM)
τ	2.339 (s)

The simulated data obtained in that way are shown on Fig. 1 (blue traces) together with the actual data (red curves) they are supposed to mimic. At a qualitative level at least, our data generation model is able to produce realistic looking simulations.

### Software and simulation details

The methodological details of the measurements to which the analysis presented in the present manuscript was applied are described in the companion paper [3].

The simulations, computations and figures of the present manuscript were done with Python 3 (https://www.python.org/), numpy (https://numpy.org/), scipy and matplotlib (https://matplotlib.org/). The Python codes and the data required to reproduce the simulations and figures presented in this manuscript can be downloaded from GitLab (https://gitlab.com/c_pouzat/getting-se-on-ratiometric-ca-estimator).

The use of scipy was kept to a bare minimum to maximize code lifeduration (scipy tends to evolve too fast with minimal concern for backward compatibility). The random number generators used were therefore the ones of numpy: the uniform random number generator derives from the Permuted Congruential Generator (64-bit, PCG64) (https://www.pcg-random.org/) [11] while the normal random number generator is an adaptation of the Ziggurat method [12] of Julia (https://docs.julialang.org/en/v1/); unfortunately one has to check the source code of both numpy and Julia to find that out.

### Are the standard errors of ratiometric estimator accurate?

Since the two ${\hat{\sigma }}_{\hat{Ca}}^{2}$ estimation methods, propagation of uncertainty and Monte-Carlo, agree at each time point within 2%, we illustrate in this section the results obtained with the Monte-Carlo method.

We take next the simulated data (blue curves on Fig. 1) together with the simulated background signals (not shown) as if they were actual data and we compute the ratiometric estimator and its standard error as described in Section 2.4, using $k=10^{4}$ replicates. Fig. 2 shows the standardized residuals as well as the simulated data together with the true [Ca${}^{2+}$], we know it since we used it to simulate the data!

The upper part of Fig. 2 is only a qualitative way of checking that the normalized residuals follow a standard normal distribution. A quantitative assessment is provided by the Shapiro-Wilk W statistic, that is here: 0.987; giving a p-value of 0.128. There is therefore no ground for rejecting the null hypothesis that the normalized residuals are IID draws from a standard normal distribution.

**Fig. 3:**
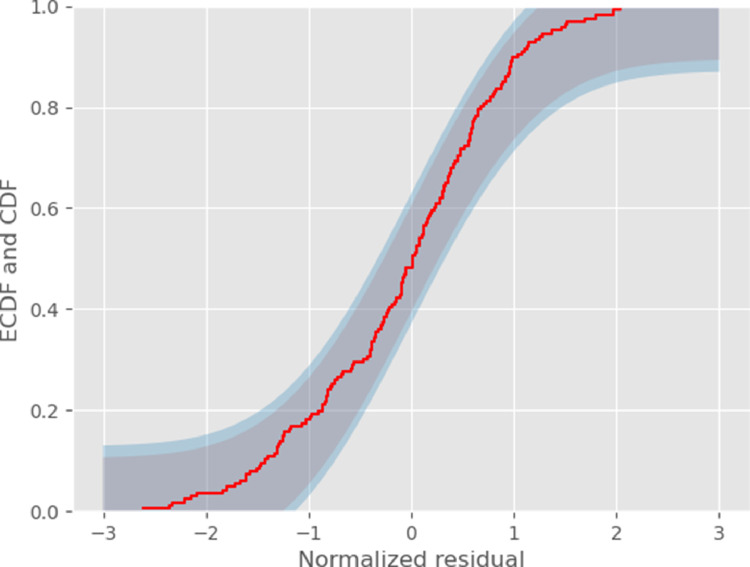
Empirical cumulative distribution function (ECDF) of the normalized residuals (red) together with 95% (grey) and 99% (blue) Kolomogorov confidence bands.

As an additional, visual but less powerful test, we plot the empirical cumulative distribution function (ECDF) of the normalized residuals together with the theoretical (normal) one and with Kolmogorov’s confidence bands (Fig. 3). If the empirical ECDF arises from a normally distributed sample with mean 0 and SD 1, it should be *completely* contained in the 95% confidence band 95% of the time and in the 99% band, 99% of the time (these are *confidence bands* not collections of pointwise confidence intervals).

We conclude from these visual representations and formal tests that our normalized residuals follow the expected standard normal distribution, implying that our proposed method for getting the standard errors of the ratiometric estimator is fundamentally correct.

## Discussion

We have presented a new and simple method for getting standard errors on calcium concentration estimates from ratiometric measurements. This method does not require any more data than what experimentalists using ratiometric dyes like Fura-2 are usually collecting: measurements at 340 and 380 nm both within a region of interest and within a background measurement region. Once the errors bars have been obtained, arbitrary models can be fitted to the calcium transients – by weighted nonlinear least-squares [10] – and meaningful confidence intervals for the parameters of these models will follow as illustrated in the companion paper [3]. The present contribution is therefore best viewed as a major simplification of the ‘direct approach’ of [1]. In contrast to the latter, the new method does not require a ‘3 wavelengths protocol’, it does not require either a precise fit of the autofluorescence dynamics at the three wavelengths and is therefore much easier to implement. We provide moreover two independent implementations, one in C and one in Python, they are open source and freely available. The rather verbose Python implementation of the heart of the method (Section 2.4) requires 25 lines of code and nothing beyond basic numpy functions. We are therefore confident that this method could help experimental physiologists getting much more quantitative results at a very modest extra cost.

## Co-submission

This manuscript is a co-submission associated with *Analysis of neuronal*$Ca^{2+}$ *handling properties by combining perforated patch clamp recordings and the added buffer approach*, DOI: 10.1016/j.ceca.2021.102411.

## Declaration of Competing Interest

The authors declare that they have no known competing financial interests or personal relationships that could have appeared to influence the work reported in this paper.

## Figures and Tables

**Fig. 4:**
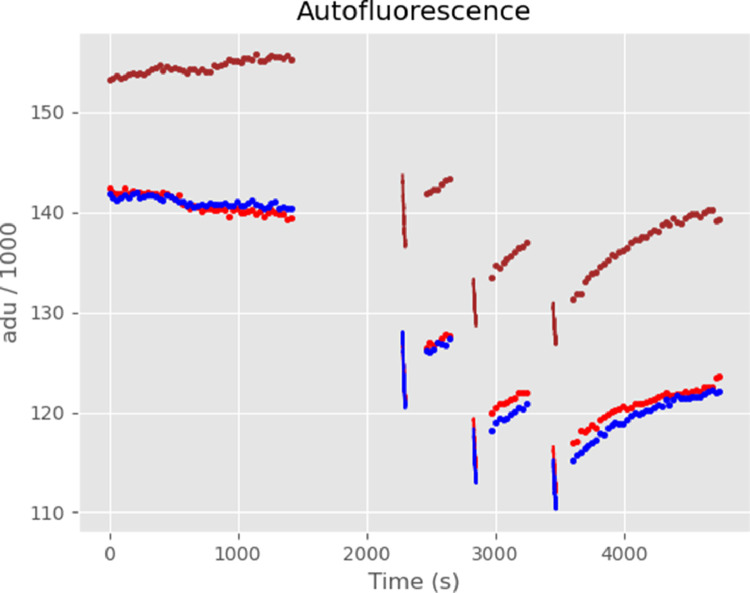
Autofluorescence at 3 excitation wavelengths, 340 nm (red), 360 (blue), 380 (brown). Both during low frequency Stimulation (four portions made of dots) and during the transients where a higher frequency stimulation was applied (3 groups with almost vertical lines).

**Fig. 5:**
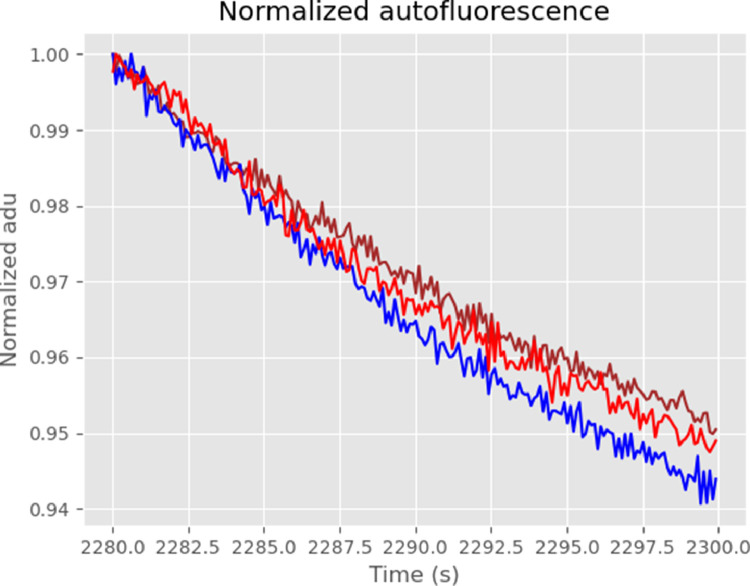
Normalized autofluorescence at 3 excitation wavelengths, 340 nm (red), 360 nm (blue) and 380 (brown) during the first transient. At each wavelength, the normalization is performed by dividing each value by the maximal one.

**Fig. 6:**
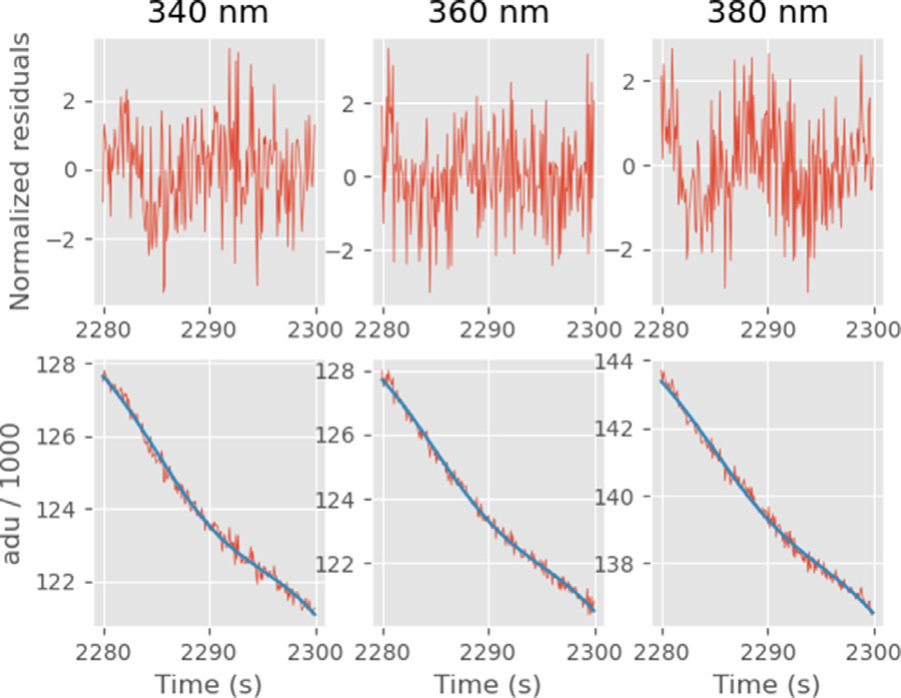
Bottom: Autofluorescence (red) at 340 nm (left), 360 nm (middle) and 380 nm (right) together with a straight line plus cosine function fit (blue). Top, the normalized residuals: $\left(\text{adu}-\text{fit}\right)/\sqrt{G\;\text{fit}+P_{B}\;G^{2}\;\sigma_{read-out}^{2}}$.

## Appendix A Propagation of uncertainty

We outline in this section how to reach Eqs. (11)–(13) of Section 2.4.1. We first need to remember that is X and Y are two *independent* random variables with mean $EX=\mu_{X}$ and $EY=\mu_{Y}$ and variance $VX=\sigma_{X}^{2}$ and $VY=\sigma_{Y}^{2}$, then if Z=a+bX+cY (a,b,c∈R) we have:

$$\begin{array}{cc} EZ & =a+b\;\mu_{X}+c\;\mu_{Y}\;,\\ VZ & =b^{2}\;\sigma_{X}^{2}+c^{2}\;\sigma_{Y}^{2}\;. \end{array}$$

Eq. (11) is a direct consequence of the last equality. If X is (approximately) normally distributed with ($X∼N(\mu_{X},\sigma_{X}^{2})$) as well as Y, we can write: $X\approx \mu_{X}+Z_{1}\sigma_{X}$ and $Y\approx \mu_{Y}+Z_{2}\sigma_{Y}$, where $Z_{1}$ and $Z_{2}$ are independent and follow a standard normal distribution, N(0,1). If now Z=f(X,Y) and the partial derivatives of f at $\left(\mu_{X},\mu_{Y}\right)$ exist then:

$$\begin{array}{cc} Z & =f(\mu_{X}+Z_{1}\sigma_{X},\mu_{Y}+Z_{2}\sigma_{Y})\\ & \approx f\left(\mu_{X},\mu_{Y}\right)+Z_{1}\sigma_{X}\frac{\partial f(\mu_{X},\mu_{Y})}{\partial X}+Z_{2}\sigma_{Y}\frac{\partial f(\mu_{X},\mu_{Y})}{\partial Y}\;. \end{array}$$

This is just a first order Taylor expansion and that is where the ‘small enough standard error’ assumption is necessary. Z is then (approximately) a linear combination of two independent standard normal random variables and we immediately get:

$$\begin{array}{cc} EZ & =f(\mu_{X},\mu_{Y})\;,\\ VZ & ={\left(\frac{\partial f(\mu_{X},\mu_{Y})}{\partial X}\right)}^{2}\;\sigma_{X}^{2}+{\left(\frac{\partial f(\mu_{X},\mu_{Y})}{\partial Y}\right)}^{2}\;\sigma_{Y}^{2}\;. \end{array}$$

Eq. (12) follows directly by computing the necessary partial derivatives, while Eq. (13) requires the computation of a single derivative.

## Appendix B Auto-fluorescence dynamics

### B1 General features

The evolution of the aduλB is shown on Fig. 4. We see that the autofluorescence runs down when high frequency flashes are applied during the 3 transients, with a partial recovery between transients.

### B2 Within transient dynamics

The ‘direct method’ of [1] requires the knowledge of the autofluorescence value at each time point during a transient at both 340, 360 and 380 nm, since Eqs. (4) and (6) are fitted directly to the recorded adu${}_{340}$ and adu${}_{380}$ and they depend on the total Fura concentration at transient time that is estimated from the difference of the 360 nm measurements in the ROI and the BMR. We therefore take a closer look a the autofluorescence dynamics during the first transient (Fig. 5).

At that stage we can fit a straight line plus a cosine function whose period is the duration of a transient. That’s a good way to capture the main structure in the transient, but is still does not account for the full signal variability (Fig. 5). As can be seen from the normalized residuals – the residuals divided by the standard deviation – that should be very nearly independent random draws from a standard normal distribution if the model is correct, there are finer structures left (like the double valley on the 380 nm residuals) meaning that those fits won’t pass formal goodness of fit tests. Indeed if we apply Pearson’s $\chi^{2}$ tests to these stabilized residuals we get:

• at 340 nm a residual sum of squares (RSS) 326, leading to a $P(\chi_{197}^{2}>326)=$ 0.0,
• at 360 nm a RSS of 288, leading to a $P(\chi_{197}^{2}>288)=$ 2.6e-05,
• at 380 nm a RSS of 275, leading to a $P(\chi_{197}^{2}>275)=$ 0.000203.

We are then left with three possibilities:

1. try to refine the ‘straight line plus cosine function’ empirical model in order to get acceptable fits,
2. try to get a better understanding of the autofluorescence dynamics,
3. find another way to get error bars on our estimates.

Since we wanted to propose an ‘as general and easy as possible’ method we chose the third approach in the present manuscript.
